# D-form KLKLLLLLKLK-NH_2_ peptide exerts higher antimicrobial properties than its L-form counterpart via an association with bacterial cell wall components

**DOI:** 10.1038/srep43384

**Published:** 2017-03-06

**Authors:** Takayuki Manabe, Kiyoshi Kawasaki

**Affiliations:** 1 Faculty of Pharmaceutical Sciences, Doshisha Women’s College, Kyotanabe, Kyoto 610-0395, Japan

## Abstract

The antimicrobial peptide KLKLLLLLKLK-NH_2_ was developed based on sapesin B, and synthesized using D-amino acids. Biochemical properties of the D-form and L-form KLKLLLLLKLK-NH_2_ peptides were compared. In order to limit the effects due to bacterial resistance to proteolysis, antimicrobial activities of the peptides were evaluated after short-term exposure to bacteria. D-form KLKLLLLLKLK-NH_2_ exhibited higher antimicrobial activities than L-form KLKLLLLLKLK-NH_2_ against bacteria, including *Staphylococcus aureus* and *Escherichia coli*. In contrast, both the D-form and L-form of other antimicrobial peptides, including Mastoparan M and Temporin A, exhibited similar antimicrobial activities. Both the D-form KLKLLLLLKLK-NH_2_ and L-form KLKLLLLLKLK-NH_2_ peptides preferentially disrupted *S. aureus*-mimetic liposomes over mammalian-mimetic liposomes. Furthermore, the D-form KLKLLLLLKLK-NH_2_ increased the membrane permeability of *S. aureus* more than the L-form KLKLLLLLKLK-NH_2_. Thus suggesting that the enhanced antimicrobial activity of the D-form was likely due to its interaction with bacterial cell wall components. *S. aureus* peptidoglycan preferentially inhibited the antimicrobial activity of the D-form KLKLLLLLKLK-NH_2_ relative to the L-form. Furthermore, the D-form KLKLLLLLKLK-NH_2_ showed higher affinity for *S. aureus* peptidoglycan than the L-form. Taken together, these results indicate that the D-form KLKLLLLLKLK-NH_2_ peptide has higher antimicrobial activity than the L-form via a specific association with bacterial cell wall components, including peptidoglycan.

Cationic antimicrobial peptides act as innate immune agents in multicellular organisms including mammals and insects (reviewed in ref. 1). Antimicrobial peptides specifically bind to negatively-charged bacterial surface molecules, such as the endotoxin lipopolysaccharide^2^,^3^,^4^, and disrupt the bacterial membrane leading to cell lysis and/or cell death. The Shai-Matsuzaki-Huang model is a well-known machinery that explains the activity of antimicrobial peptides^1^,^5^,^6^,^7^. Antimicrobial peptides have attracted great interest as a novel class of antibiotics because they exhibit broad-spectrum antimicrobial activities and they are not likely to induce resistance. Unfortunately, the susceptibility of antimicrobial peptides to proteases limits their use in pharmaceutical applications. To overcome this limitation, antimicrobial peptides may be synthesized from D-amino acids instead of L-amino acids, which makes them resistant to proteolytic degradation.

Both D-form and L-form antimicrobial peptides showed similar antimicrobial activity^8^,^9^,^10^,^11^,^12^. However, D-form antimicrobial peptides were more stable and retained antimicrobial activity for extended periods of time compared to L-form antimicrobial peptides in the presence of NaCl, CaCl_2_, or human serum albumin at physiological concentrations^11^, and D-form antimicrobial peptides were resistant to enzymatic degradation^10^. Furthermore, a D-form 13-mer antimicrobial peptide, but not the L-form 13-mer antimicrobial peptide, reduced endotoxin-induced lethality in mice^8^. The D-form-specific effect was explained by protease resistance *in vivo*^9^,^12^. Furthermore, antimicrobial peptides were thought to exert their effects without specific target receptors which require close contact based on the structure of the peptides^10^,^12^.

Sapecin B is an antimicrobial peptide that was originally isolated from the culture medium of an embryonic cell line, NIH-Sape-4, derived from *Sarcophaga peregrine* (flesh fly). It displays potent activity against Gram-positive bacteria^13^. Two other related proteins, sapecin and sapecin C, were also isolated from culture medium of NIH-Sape-4^13^,^14^,^15^. Sapecin B has significant sequence similarity to a scorpion venom toxin, charybdotoxin^13^,^16^. Structural comparison of sapecin B and charybdotoxin identified the undecapeptide RSLCLLHCRLK-NH_2_, which corresponds to amino acid residues 7 to 17 of sapecin B with C-terminal amidation^16^,^17^. The peptide fragment RSLCLLHCRLK-NH_2_ showed significant antimicrobial activity, suggesting that this region is responsible for the antimicrobial activity of the peptide^17^. The undecapeptide KLKLLLLLKLK-NH_2_ was developed by modifying the primary structure of RSLCLLHCRLK-NH_2_. In addition to its activity against Gram-positive bacteria, Gram-negative bacteria, and fungi^18^, KLKLLLLLKLK-NH_2_ has been shown to enhance mammalian immune responses via undefined molecular mechanisms^19^,^20^,^21^. The antimicrobial activity of the D-form KLKLLLLLKLK-NH_2_, which was synthesized using D-amino acids, persisted longer than the L-form because of its resistance to proteolytic degradation^18^.

In this study, we examined the antimicrobial properties of D-form KLKLLLLLKLK-NH_2_. D-form KLKLLLLLKLK-NH_2_ displays higher antimicrobial activity against bacteria than its L-form; however, this elevated activity could not be explained by resistance to proteolytic degradation. It is important to note that other D-form antimicrobial peptides did not show higher antimicrobial activity than their L-form counterparts. Furthermore, D-form KLKLLLLLKLK-NH_2_ showed higher affinity for bacterial cell wall components, such as peptidoglycan, than its L-form. Thus, the enhanced antimicrobial activity of the D-form KLKLLLLLKLK-NH_2_ relative to its L-form is due to direct interactions with bacterial cell surface components.

## Results

### MICs of D-form KLKLLLLLKLK-NH_2_ were lower than those of L-form KLKLLLLLKLK-NH_2_

Previously, D-form KLKLLLLLKLK-NH_2_ was shown to persist longer in bacterial culture medium and it showed higher antimicrobial activity to *Staphylococcus aureus* than the L-form^18^. In order to further examine the antimicrobial properties of D-form KLKLLLLLKLK-NH_2_, we determined the MICs of the peptides against *S. aureus, Escherichia coli*, and *Candida albicans*. MICs of D-form KLKLLLLLKLK-NH_2_ were lower than those of its L-form, especially against *S. aureus* where the MIC of the D-form was 16-fold lower than the L-form (Table 1). We determined minimum inhibitory concentrations (MICs) of other antimicrobial peptides, including KLKLLLKLK-NH_2_, a derivative of KLKLLLLLKLK-NH_2_^18^, FIKRIARLLRKIF-NH_2_ (Kn2–7) derived from *Buthus martensii* scorpion venom^22^, INLKAIAALAKKLL-NH_2_ (Mastoparan M) derived from *hornet venom*^23^, and FLPLIGRVLSGIL-NH_2_ (Temporin A) derived from *Rana temporariareference*^24^ against *S. aureus*. All of these peptides are expected to form a helical structure similar to KLKLLLLLKLK-NH_2_^16^,^17^,^22^,^23^,^24^. MIC of D-form KLKLLLKLK-NH_2_ against *S. aureus* is more than 32-fold lower than that of the L-form (Table 2). In contrast, the MIC of D-forms and L-forms of Mastoparan M, Kn2–7, and Temporin A against *S. aureus* (Table 2) were similar. These observations indicate that KLKLLLLLKLK-NH_2_ and its related peptide KLKLLLKLK-NH_2_ are unique because these D-form peptides display lower MICs against *S. aureus* than their L-forms.

### D-form KLKLLLLLKLK-NH_2_ displayed higher antimicrobial activity than L-form KLKLLLLLKLK-NH_2_

**Figure 1:**
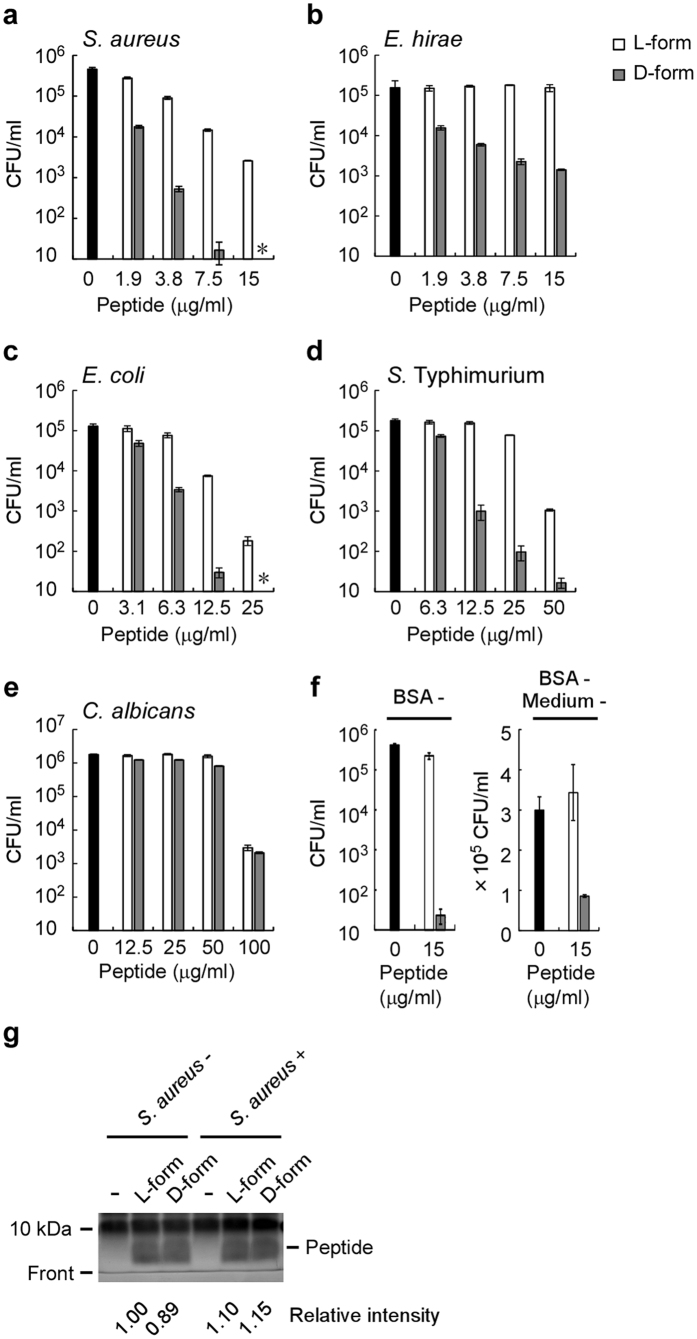
Antimicrobial activities of D-form KLKLLLLLKLK-NH_2_ to bacteria were higher than those of the L-form counterpart. Antimicrobial activities of D-form KLKLLLLLKLK-NH_2_ (D-form) and L-form KLKLLLLLKLK-NH_2_ (L-form) against *S. aureus* (**a**,**f**), *E. hirae* (**b**), *E. coli* (**c**), *S.* Typhimurium (**d**), and *C. albicans* (**e**) were examined. (**f**) Antimicrobial activities were examined in the absence of bovine serum albumin (BSA-) or in the absence of both bovine serum albumin and culture medium (BSA-, medium-). Gray bars and white bars represent CFUs in assay mixtures treated with the indicated concentrations of D-form and L-form peptides, respectively. Black bars represent CFUs in assay mixtures without peptide. The error bars represent the mean ± standard deviations from triplicate plates. Asterisk (*) indicates no bacteria were detected (<10 CFU/ml). Concentrations of dimethyl sulfoxide in the assay mixtures were 0.15% (**a,b** and **f**) and 1.0% (**c–e**). (**g**) *S. aureus (S. aureus*+) was treated without (-) or with 15 μg/ml D-form KLKLLLLLKLK-NH_2_ (D-form) or L-form KLKLLLLLKLK-NH_2_ (L-form) as performed in (**a**). Control assay mixtures without *S. aureus (S. aureus*-) were also prepared. The assay mixtures (10 μl) were subjected to SDS-polyacrylamide gel electrophoresis using Any kD^TM^ precast polyacrylamide gel. Proteins, including KLKLLLLLKLK-NH_2_ (molecular weight 1321.8), were visualized with Ruby protein gel stain. The full-length gel was shown in supplementary figure 1. Relative intensities of peptide signals were measured using Multi Gauge ver.3 (FUJIFILM), and the values normalized with that of L-form peptide (*S. aureus*-) were indicated.

**Figure 2:**
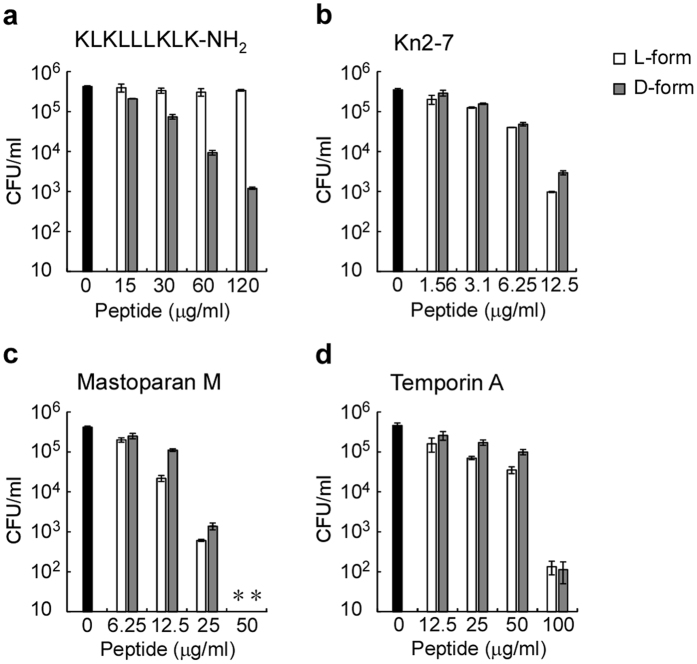
Similar antimicrobial activities of D-forms and L-forms of Kn2–7, Mastoparan M, and Temporin A. Antimicrobial activities of KLKLLLKLK-NH_2_ (**a**), Kn2–7 (**b**), Mastoparan M (**c**), and Temporin A (**d**) against *S. aureus* were examined. Gray bars and white bars represent CFUs in assay mixtures treated with indicated concentrations of D-form and L-form peptides, respectively. Black bars represent CFUs in assay mixtures treated without peptide. The error bars represent the mean ± standard deviations from triplicate plates. Asterisk (*) indicates bacteria were not detected (less than 10 CFU/ml). Concentrations of dimethyl sulfoxide in the assay mixtures were 0. 5%.

In order to further explorer the antimicrobial properties of D-form KLKLLLLLKLK-NH_2_, we incubated microorganisms with the peptides for 10 min to minimize the effect of proteolytic degradation. D-form KLKLLLLLKLK-NH_2_ showed higher antimicrobial activity to *S. aureus, Enterococcus hirae, E. coli*, and *Salmonella enterica* serovar Typhimurium than L-form KLKLLLLLKLK-NH_2_ (Fig. 1a–d). In contrast, D-form and L-form KLKLLLLLKLK-NH_2_ showed similar antimicrobial activity against *C. albicans* (Fig. 1e). Under the experimental conditions in which we examined antimicrobial activity against *S. aureus*, specific degradation of the L-form peptide was not observed (Fig. 1g). This observation suggests that the higher antimicrobial activity of D-form KLKLLLLLKLK-NH_2_ was not due to its resistance to proteolytic degradation. In addition, in order to exclude the possibility that bovine serum albumin or some components from culture medium specifically affect the antimicrobial activity of KLKLLLLLKLK-NH_2_, we performed experiments without culture medium and/or bovine serum albumin in the assay mixture. D-form KLKLLLLLKLK-NH_2_ also showed higher antimicrobial activity to *S. aureus* than L-form KLKLLLLLKLK-NH_2_ in the absence of culture medium and/or bovine serum albumin (Fig. 1f). It is noteworthy that antimicrobial activity of both L-form and D-form peptide in the absence of culture medium and bovine serum albumin were lower than those in our standard assay condition (Fig. 1a and f). The antimicrobial activity of D-form KLKLLLKLK-NH_2_ was also higher than that of its L-form counterpart (Fig. 2a). In contrast, the D-forms and L-forms of Kn2–7, Mastoparan M, and Temporin A peptides displayed similar antimicrobial activities against *S. aureus* (Fig. 2b–d). These results indicate that KLKLLLLLKLK-NH_2_ and its derivative KLKLLLKLK-NH_2_ are unique in that their D-forms have antimicrobial activities than their L-forms.

### D-form KLKLLLLLKLK-NH_2_ increased bacterial membrane permeability

**Figure 3:**
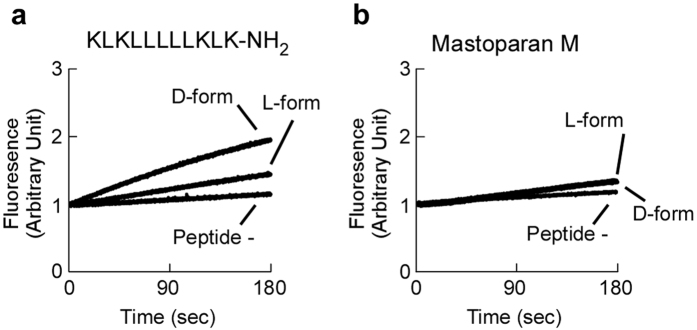
Effect of D-form KLKLLLLLKLK-NH_2_ on *S. aureus* membrane permeability was higher L-form KLKLLLLLKLK-NH_2_. (**a**) *S. aureus* was treated with 20 μg/ml of D-form KLKLLLLLKLK-NH_2_, 20 μg/ml of L-form KLKLLLLLKLK-NH_2_, or without peptide (peptide-). (**b**) *S. aureus* was treated with 20 μg/ml D-form Mastoparan M, 20 μg/ml L-form Mastoparan M, or without peptide (peptide-). Ethidium influx was monitored by fluorescence for 180 sec after the addition of ethidium bromide to the bacteria/peptide suspension.

Cationic antimicrobial peptides bind to the negatively charged bacterial surface and penetrate into the bacterial membrane. Therefore, their effects on bacterial membrane permeability closely correlate with antimicrobial activity. Effects of KLKLLLLLKLK-NH_2_ and Mastoparan M on membrane permeability of *S. aureus* were monitored by ethidium bromide influx rates. As shown in Fig. 3a, both D-form KLKLLLLLKLK-NH_2_ (20 μg/ml) and L-form KLKLLLLLKLK-NH_2_ (20 μg/ml) increased ethidium bromide influx rates; however, the rates were higher in response to D-form KLKLLLLLKLK-NH_2_ than the L-form KLKLLLLLKLK-NH_2_. In contrast, D-form and L-form Mastoparan M (20 μg/ml) increased ethidium bromide influx rates to a similar extent (20 μg/ml) (Fig. 3b). These observations are consistent with the findings that the antimicrobial activity of D-form KLKLLLLLKLK-NH_2_ against *S. aureus* was higher than that of its L-form KLKLLLLLKLK-NH_2_ (Fig. 1a). However, that antimicrobial activity of D-form Mastoparan M against *S. aureus* was similar with that of its L-form (Fig. 2c).

### *S. aureus* peptidoglycan and *E. coli* lipopolysaccharide preferentially inhibited the antimicrobial activity of D-form KLKLLLLLKLK-NH_2_

**Figure 4:**
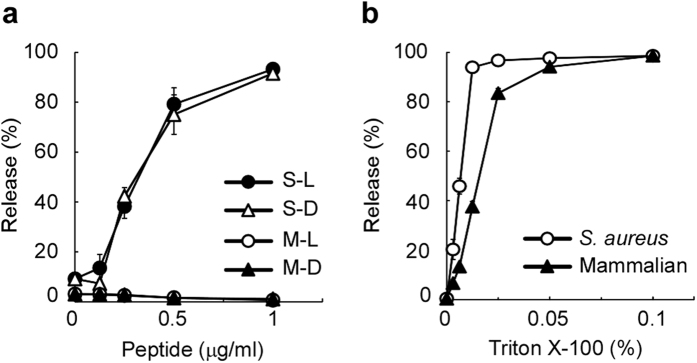
D-form and L-form KLKLLLLLKLK-NH_2_ disrupt *S. aureus*-mimetic liposomes. (**a**) *S. aureus*-type and mammalian-type liposomes with calcein were exposed to D-form and L-form KLKLLLLLKLK-NH_2_: S-L, *S. aureus*-mimetic liposomes treated with L-form peptide; S-D, *S. aureus*-mimetic liposomes treated with D-form peptide; M-L, mammalian-mimetic liposomes treated with L-form peptide; and M-D, mammalian-mimetic liposomes treated with D-form peptide. (**b**) *S. aureus*-type (*S. aureus*) and mammalian-type (Mammalian) liposomes containing calcein were exposed to Triton X-100. The amount of calcein that leaked from the liposomes was measured using a spectrofluorophotometer and normalized to determine the % release relative to 0.1% Triton X-100. The error bars represent the mean ± standard deviations from triplicate assays.

Most cationic antimicrobial peptides interact with bacterial membranes. Previously, sapecin was shown to have a high affinity for cardiolipin^25^. This observation encouraged us to examine whether D-form KLKLLLLLKLK-NH_2_ specifically disrupts liposomes that mimic the cellular membrane of *S. aureus*. Both D-form and L-form KLKLLLLLKLK-NH_2_ released calcein from *S. aureus*-mimetic liposomes^17^,^26^, which consisted of phosphatidylglycerol and cardiolipin (Fig. 4a). On the other hand, neither D-form nor L-form KLKLLLLLKLK-NH_2_ was able to release calcein from mammalian-mimetic liposomes^27^ that consisted of phosphatidylcholine, phosphatidylethanolamine, and cholesterol (Fig. 4a). Mammalian-mimetic liposomes demonstrated similar sensitivity to Triton X-100 as *S. aureus*-mimetic liposomes, excluding the possibility that mammalian-mimetic liposome are resistant to chemical treatments (Fig. 4b). These observations indicate that both D-form and L-form KLKLLLLLKLK-NH_2_ preferentially disrupt *S. aureus*-mimetic liposomes, which likely contributes to the antimicrobial activity of KLKLLLLLKLK-NH_2_. Thus, the ability to disrupt *S. aureus*-mimetic liposomes is not the cause of higher antimicrobial activity of D-form KLKLLLLLKLK-NH_2_ relative to its L-form.

**Figure 5:**
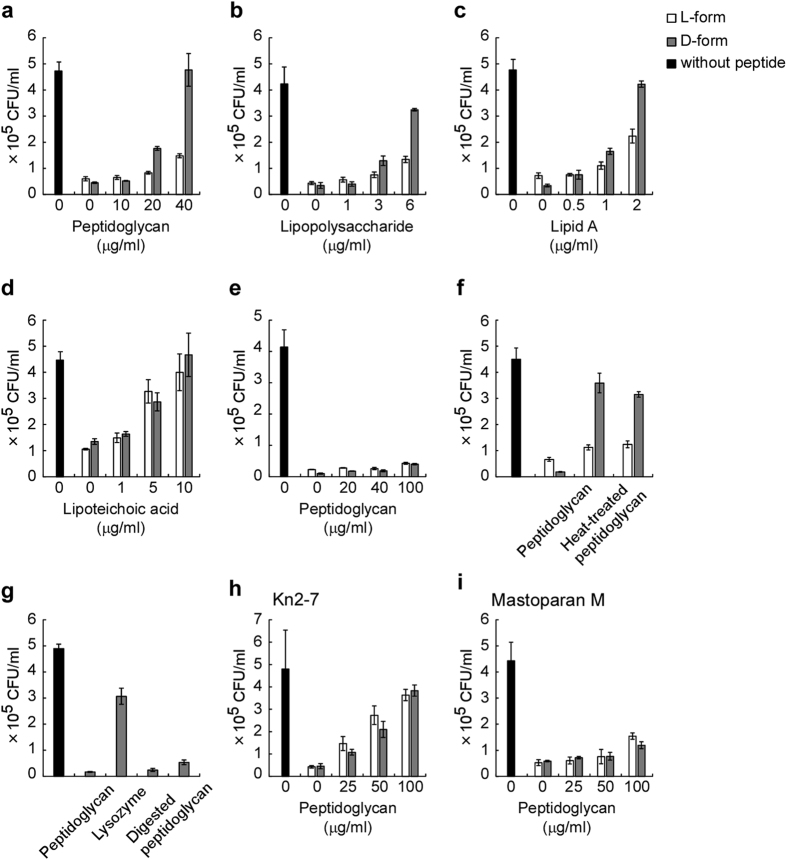
*S. aureus* peptidoglycan and *E. coli* lipopolysaccharide preferentially inhibit the antimicrobial activity of D-form KLKLLLLLKLK-NH_2_. Antimicrobial activities of D-form KLKLLLLLKLK-NH_2_ (1.9 μg/ml) and L-form KLKLLLLLKLK-NH_2_ (7.5 μg/ml) against *S. aureus* were examined in the presence of the indicated concentrations of peptidoglycan from *S. aureus* (**a**), lipopolysaccharide from *E. coli* (**b**), lipid A (**c**), lipoteicoic acid from *S. aureus* (**d**), and peptidoglycan from *E. coli* (**e**). (**f**) Antimicrobial activities of D-form KLKLLLLLKLK-NH_2_ (1.9 μg/ml) and L-form KLKLLLLLKLK-NH_2_ (7.5 μg/ml) against *S. aureus* were examined in the absence or presence of peptidoglycan (40 μg/ml) or heat-treated peptidoglycan (40 μg/ml) from *S. aureus*. (**g**) Antimicrobial activities of D-form KLKLLLLLKLK-NH_2_ (2.0 μg/ml) against *S. aureus* were examined in the absence or presence of 40 μg/ml of peptidoglycan treated with lysozyme (digested peptidoglycan), 40 μg/ml of peptidoglycan treated without lysozyme (peptidoglycan), or control buffer treated with lysozyme (lysozyme). Antimicrobial activities of D-form and L-form peptides of Kn2–7 (6.25 μg/ml) (**h**) or Mastoparan M (8 μg/ml) (**i**) against *S. aureus* were examined in the presence of the indicated concentrations of peptidoglycan from *S. aureus*. Gray bars and white bars represent CFUs in assay mixtures treated with D-form and L-form peptides, respectively. Black bars represent CFU in assay mixtures treated without peptide. The error bars represent the mean ± standard deviations from triplicate plates. Concentrations of dimethyl sulfoxide in the assay mixtures were 0.15%.

To identify a specific target of D-form KLKLLLLLKLK-NH_2_, we analyzed whether bacterial cell wall components were able to inhibit the antimicrobial activities. A comparison of the antimicrobial activities of D-form and L-form KLKLLLLLKLK-NH_2_ revealed that 1.9 μg/ml of D-form and 7.5 μg/ml of L-form displayed similar antimicrobial activity to *S. aureus*. The antimicrobial effect of D-form KLKLLLLLKLK-NH_2_ was almost inhibited by 40 μg/ml of *S. aureus* peptidoglycan, but the same concentration failed to abrogate the antimicrobial activity of L-form (Fig. 5a). These observations highlight the potential for a specific interaction between D-form KLKLLLLLKLK-NH_2_ and peptidoglycan. In order to exclude the possibility that some contaminants, such as proteases, in peptidoglycan samples might affect the inhibitory effects, heat-treated peptidoglycan was used for the analysis. As shown in Fig. 5f, heat-treated peptidoglycan showed similar inhibitory effects on the antimicrobial activities with those of untreated peptidoglycan. To further confirm that peptidoglycan is a specific target of D-form KLKLLLLLKLK-NH_2_, the antimicrobial effects were investigated in the presence of lysozyme-digested peptidoglycans (Fig. 5g). The D-form did not show an inhibitory effect on antimicrobial activity. The antimicrobial activity of D-form KLKLLLLLKLK-NH_2_ was preferentially inhibited by lipopolysaccharide prepared from *E. coli* (Fig. 5b). Furthermore, antimicrobial activity of D-form KLKLLLLLKLK-NH_2_ was also preferentially inhibited by synthetic *E. coli* lipid A, a membrane anchor region of lipopolysaccharide (Fig. 5c). In contrast, lipoteichoic acid prepared from *S. aureus* inhibited the antimicrobial effect of both D-form and L-form peptides similarly, indicating that the inhibitory effect was not specific for the D-form peptide (Fig. 5d). Peptidoglycan prepared from *E. coli* had a weak inhibitory effect on the antimicrobial activity of D-form and L-form peptides (Fig. 5e). Taken together, these observations indicate that some cell surface components, such as *S. aureus* peptidoglycan, preferentially associate with D-form KLKLLLLLKLK-NH_2_ rather than its L-form. Moreover, this preferential association accounts for higher antimicrobial activity of D-form KLKLLLLLKLK-NH_2_ than that of the L-form.

In addition, inhibitory effects of peptidoglycan against Kn2–7 and Mastoparan M were examined. As shown in Fig. 5h, peptidoglycan shows significant inhibitory effects against the antimicrobial activities of both D-forms and L-forms of Kn2–7. Furthermore, peptidoglycan shows weak inhibitory effects to antimicrobial activities of Mastoparan M, and the inhibitory effect was not specific for the D-form peptide (Fig. 5i).

### D-form KLKLLLLLKLK-NH_2_ showed higher affinity for *S. aureus* peptidoglycan than L-form KLKLLLLLKLK-NH_2_

**Figure 6:**
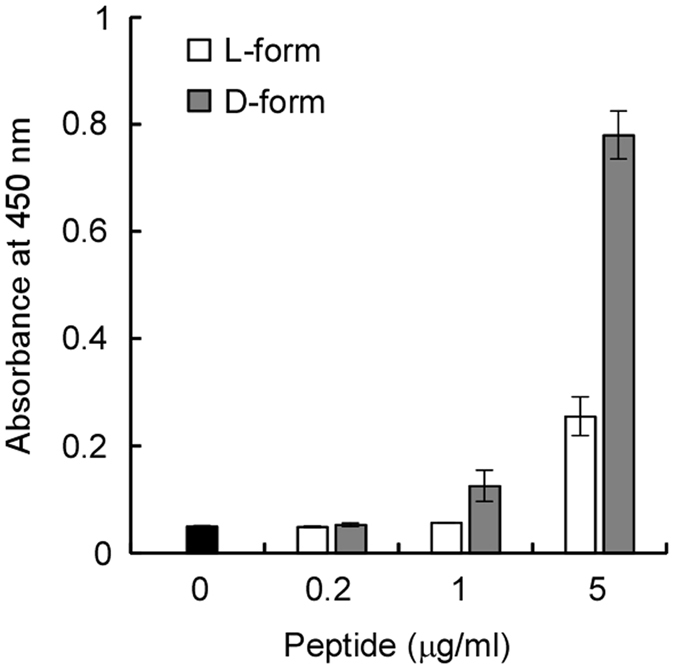
D-form KLKLLLLLKLK-NH_2_ showed higher affinity for peptidoglycan than L-form KLKLLLLLKLK-NH_2_. Peptidoglycan-coated wells were treated without (black bar) or with the indicated concentrations of biotin-labeled D-form KLKLLLLLKLK-NH_2_ (gray bar) or biotin-labeled L-form KLKLLLLLKLK-NH_2_ (white bar). Binding of biotin-labeled peptide to peptidoglycan was determined based on avidin-labeled peroxidase activity. The mean values with standard deviations of triplicate assays are shown.

The inhibitory effect of *S. aureus* peptidoglycan on the antimicrobial activity of D-form KLKLLLLLKLK-NH_2_ suggested a specific interaction between these two molecules. To determine whether there was a direct association, direct binding between KLKLLLLLKLK-NH_2_ and *S. aureus* peptidoglycan was examined. Biotin-labeled D-form or L-form KLKLLLLLKLK-NH_2_ was added to multi-well plates that were coated with immobilized *S. aureus* peptidoglycan. Binding of biotin-labeled peptides to the D-form or L-form KLKLLLLLKLK-NH_2_ was quantified using avidin-labeled peroxidase. As shown in Fig. 6, D-form KLKLLLLLKLK-NH_2_ has a higher affinity for *S. aureus* peptidoglycan than the L-form counterpart.

## Discussion

Incorporation of D-amino acids into antimicrobial peptides has been shown to improve their therapeutic efficacy; however, little is known about how the underlying mechanisms make them distinct from their L-form counterparts (reviewed in ref. 28). In this study we found that D-form KLKLLLLLKLK-NH_2_ showed higher antimicrobial activity against both Gram-positive and Gram-negative bacteria, including *S. aureus* and *E. coli*, relative to its L-form counterpart. Moreover, the enhanced antimicrobial activity of the D-form was not due to its resistance to proteolytic degradation. D-form KLKLLLLLKLK-NH_2_ showed higher affinity for *S. aureus* peptidoglycan than the L-form counterpart. Peptidoglycan and lipopolysaccharide prepared from *S. aureus* and *E. coli*, respectively, selectively inhibited the antimicrobial activities of D-form KLKLLLLLKLK-NH_2_. Thus, specific interactions between D-form peptides and components of the bacterial cell wall may contribute to its elevated antimicrobial activity.

Cationic antimicrobial peptides target the negatively charged cell surface of microorganisms. In some cases, D-forms of naturally-occurring antimicrobial peptides have antimicrobial activities similar to those of L-form counterparts, and it is believed that the interaction between antimicrobial peptide and microbial cell surface is not due to specific, close interactions^10^,^12^. This general notion is consistent with our observations of similar antimicrobial activities of the D-forms and L-forms of Mastoparan M, Kn2–7, and Temporin A. In addition, D-form KLKLLLLLKLK-NH_2_ showed similar activity to disrupt *S. aureus*-mimetic liposomes when compared to the L-form. These observations indicate that the interaction between antimicrobial peptides and anionic bacterial-type liposomes does not require close contact based on the structure, but charge-based interactions are important for antimicrobial activities. In contrast to the previous studies, our results showed that D-form KLKLLLLLKLK-NH_2_ had a higher affinity for some cell surface compounds than its L-form counterpart, and the affinity of the D-form for bacterial surface components contributed to its antimicrobial activity. Our observations indicated that some specific, close contact between antimicrobial peptides and bacterial cell surface components increase antimicrobial activities in addition to charge-based contact. Peptidoglycan is consisted of sugars and peptides, and they are chiral components. The chiral portions of peptidoglycan might be involved in the association of D-form KLKLLLLLKLK-NH_2_. It is noteworthy that high affinity of D-form KLKLLLLLKLK-NH_2_ to cell surface components including peptidoglycan does not necessary indicate direct targeting. There might be mechanisms to facilitate peptide transfer to the plasma membrane, which determine the effective concentration.

Comparison of the D-4Leu and L-4Leu antimicrobial peptides revealed that the D-form had a greater tendency to bind to the biofilm exopolysaccharide alginate^29^. This current study of KLKLLLLLKLK-NH_2_ largely recapitulated these findings. To date, the molecular basis for the close interaction of D-form peptides with bacterial cell surface components remains unknown; however, the importance of precise structures of the bacterial molecules involved in these interactions has been shown. Antimicrobial activities of D-form KLKLLLLLKLK-NH_2_ were preferentially inhibited by *S. aureus* peptidoglycan but not by *E. coli* peptidoglycan. This difference is likely based on the structural differences between *S. aureus* peptidoglycan and *E. coli* peptidoglycan.

Based on our observations, replacement of all L-amino acids with D-amino acids in an antimicrobial peptide may introduce structural changes that are beneficial for antimicrobial activity. It is important to note that not all antimicrobial peptides have distinct activities based on whether they are expressed as a D-form or L-form, and the number of these peptides may be fairly low. Future studies should focus on elucidating the specific interactions of the D-form modification with bacteria as well as the molecular basis underlying this this phenomenon. This will aid in the development of peptide therapeutics.

## Methods

### Reagents and antimicrobial peptides

Dimethyl sulfoxide, bovine serum albumin (fraction V), cardiolipin, L-α-phosphatidyl-DL-glycerol, peptidoglycan purified from *S. aureus*, lysozyme, and lipoteichoic acid purified from *S. aureus* were purchased from Sigma-Aldrich. Cholesterol, 2-dioleoyl-*sn*-glycero-3-phosphocholine, and 2-dioleoyl-*sn*-glycero-3-phosphoethanolamine were purchased from Avanti Polar Lipids Inc. Peptidoglycan purified from *E. coli* was purchased from InvivoGen. Calcein was purchased from Dojindo. Triton X-100 was purchased from Thermo Fisher Scientific. Lipopolysaccharide purified from *E. coli* 0111:B4 was purchased from List Biological Laboratories, Inc. Synthetic lipid A was purchased from Peptide Institute Inc. Ruby protein gel stain and Any kD^TM^ precast polyacrylamide gels were purchased from Bio-Rad.

Antimicrobial peptides and biotin-labeled antimicrobial peptides were commercially synthesized by Hayashi Kasei, Thermo Fisher Scientific, and the Toray Research Center. C-terminals of the synthetic peptides were modified by amidation. All peptides were initially suspended in dimethyl sulfoxide.

### Culture of bacteria and fungi

*S. aureus* (NBRC100910), *E. coli* W3110 (NBRC12713), *E. hirae* (NBRC3181), *C. albicans* (NBRC1385), and *S.* Typhimurium 14028s (ATCC14028)^2^ were used in this study. *S. aureus, E. coli*, and *S.* Typhimurium were grown at 37 °C with aeration in Muller-Hinton II medium (BD Biosciences). *E. hirae* was grown at 37 °C in LB medium (Nacalai Tesque, Inc.) containing 0.5% glucose (w/v). *C. albicans* was grown at room temperature with aeration in YM medium (BD Biosciences). All experiments were conducted using bacterial cells and *C. albicans* in the logarithmic-phase of growth.

### Determination of MIC

Bacterial suspensions in Muller-Hinton II medium were adjusted to an optical density of 550 nm (OD_550_) = 0.0011. *C. albicans* suspensions in YM medium were adjusted to OD_650_ = 0.033. Peptides were serially diluted in 10 mM phosphate buffer (pH 6.0) containing 130 mM sodium chloride, 0.2% bovine serum albumin, and 2.56% dimethyl sulfoxide. The peptide solution (100 μl) was mixed with 100 μl of bacteria or *C. albicans* suspensions. Bacterial cultures were incubated for one day at 37 °C. *C. albicans* cultures were incubated for two days at room temperature. Cell growth was monitored optically and the MIC was determined.

### Assay for antimicrobial activity

Bacteria and *C. albicans* were suspended in growth medium. Peptides suspended in dimethyl sulfoxide were serially diluted in 10 mM phosphate buffer (pH 6.0) containing 130 mM sodium chloride, 0.2% bovine serum albumin as described previously^13^. Concentrations of dimethyl sulfoxide in the assay mixtures are indicated in the figure legends. In order to examine the effects of bovine serum albumin on the assay, 10 mM phosphate buffer (pH 6.0) containing 130 mM sodium chloride, was used for the dilution of peptides. Peptide solution (500 μl) was added to 500 μl of bacteria suspensions and then the mixture was incubated at 37 °C for 10 min. In order to examine the effects of culture medium components on the assay, bacteria suspension was prepared with 10 mM phosphate buffer (pH 6.0) containing 130 mM sodium chloride. Alternatively, 500 μl of peptide solution was added to 500 μl of *C. albicans* suspensions and the mixture was incubated at room temperature for 10 min. The inhibitory effects of the bacterial components were analyzed by incubating 450 μl of *S. aureus* suspension with 500 μl of peptide solution plus 50 μl of inhibitor samples at 37 °C for 10 min. Then, the peptide/bacteria suspensions were diluted and plated onto LB agar, LB agar containing 0.5% glucose, or YM agar. After cultivation of the plates, colony forming units (CFU) in the peptide/bacteria suspension were calculated based on the average of triplicate plates.

### Assay for membrane permeability

To examine membrane permeability, ethidium influx rates were examined as previously described^30^,^31^. *S. aureus* suspension cultures were adjusted to an OD_600_ of 0.4 in 10 mM phosphate buffer (pH 6.0) containing 130 mM sodium chloride and 0.2% bovine serum albumin. Then, peptide in dimethyl sulfoxide (8 μl) or dimethyl sulfoxide alone (8 μl) was added to 2 ml of *S. aureus* suspensions. At 30 sec after the addition of peptide, ethidium bromide was added to a final concentration of 5 μg/ml, and fluorescence of the ethidium-nucleic acid complex was monitored using a RF-5300PC spectrofluorometer (Shimadzu). Excitation and emission wavelengths were 545 nm with 5 nm slits and 600 nm with 10 nm slits, respectively.

### Preparation of liposomes containing calcein and assay for resistance to antimicrobial peptides

Liposomes were prepared as previously described with some modifications^18^,^32^. *S. aureus-*mimetic liposomes^17^,^26^ and mammalian-mimetic liposomes^27^ were prepared by combining phospholipid mixtures (cardiolipin: L-α-phosphatidyl-DL-glycerol = 1:3 (mol/mol)) and 2-dioleoyl-*sn*-glycero-3-phosphocholine:1,2-dioleoyl-*sn*-glycero-3-phosphoethanolamine:Cholesterol = 4:2:3 (mol/mol/mol)), respectively. A 50 mM calcein solution was prepared by mixing 100 mg calcein with 3.1 ml of solution A (0.3 ml of 1 M Tris-HCl (pH 7.4), 0.9 ml of 5 M sodium hydroxide, and 13.8 ml of water). The liposomes were prepared by adding 1 ml of calcein solution to the dried lipid mixtures, followed by vortexing for 10 min at room temperature. The liposome solution (1 ml) was centrifuged at 3000 × *g* for 10 min, and the precipitate was resuspended in 1 ml of 20 mM Tris-HCl (pH 7.4) containing 150 mM sodium chloride. The centrifugation step was repeated two more times. Finally, the precipitate was suspended in 100 μl of 20 mM Tris-HCl (pH 7.4) containing 150 mM sodium chloride.

Liposome suspensions were prepared by diluting of 1 μl of liposomes into 40 ml of 10 mM phosphate buffer (pH6.0) containing 130 mM sodium chloride. Peptides were serially diluted in 10 mM phosphate buffer (pH 6.0) containing 130 mM sodium chloride and 1% dimethyl sulfoxide. Peptide samples (20 μl) were added to 2 ml of liposome suspension, and the mixtures were incubated at room temperature for 10 min. Calcein leakage from the liposomes was examined using a RF-5300PC spectrofluorometer. Excitation and emission wavelengths were 490 nm and 520 nm (with a 5 nm slit width), respectively^32^.

### Digestion and heat-inactivation of peptidoglycan

Peptidoglycan (120 μg) prepared from *S. aureus* was added to 1 mg/ml of lysozyme in phosphate buffered saline (150 μl)^33^. Peptidoglycan without lysozyme and lysozyme without peptidoglycan were also prepared as controls. Samples were incubated overnight at 37 °C, and then incubated at 100 °C for 15 min to inactivate lysozyme. For heat-inactivation of peptidoglycan, 600 μg of peptidoglycan suspended in water (300 μl) was incubated at 100 °C for 15 min. The samples were sonicated for 10 sec at setting 1 using a Branson sonifier model S-150D. These samples were used as inhibitor samples for antimicrobial activity assays.

### Peptidoglycan-binding assay

Peptidoglycan-binding assays were performed as previously described with some modifications^34^,^35^,^36^. Peptidoglycan from *S. aureus* (100 μg/ml) was suspended in 0.2% trifluoroacetic acid and sonicated twice for 10 sec at setting 1 using a Branson sonifier model S-150D. The peptidoglycan suspension (50 μl) was used to coat the wells of a flat bottom 96-well microplate (Thermo Fisher Scientific). The plate was incubated at room temperature until the water evaporated. The plate was placed at 60 °C for 1 h to dry out completely, and then blocked with 200 μl of 5 mg/ml bovine serum albumin in binding buffer (10 mM phosphate buffer (pH 6.0) containing 130 mM sodium chloride, 0.05% Tween 20, and 0.01% trifluoroacetic acid) at 37 °C for 2 h. The plate was washed four times with 200 μl of binding buffer. Biotin-labeled peptides in 100 μl of binding buffer containing 0.5% dimethyl sulfoxide were added to the wells and incubated at 37 °C for 2 h. Detection of biotin-labeled peptides was performed using Vectastain ABC reagent (Vector Laboratories) according to manufacturer’s instructions. The wells were washed four times with binding buffer, then 100 μl of avidin-labeled peroxidase was added to each well, and the plate was incubated at 37 °C for 1 h. The wells were washed again as described above. After washing, 100 μl of 3, 3′, 5, 5′-tetramethylbenzide substrate was added and the plate was incubated at room temperature. After 10 min, the reaction was stopped by the addition of 100 μl of 0.5 M sulfuric acid. Absorbance was measured at 450 nm.

## Additional Information

**How to cite this article:** Manabe, T. and Kawasaki, K. D-form KLKLLLLLKLK-NH_2_ peptide exerts higher antimicrobial properties than its L-form counterpart via an association with bacterial cell wall components. *Sci. Rep.* **7**, 43384; doi: 10.1038/srep43384 (2017).

**Publisher's note:** Springer Nature remains neutral with regard to jurisdictional claims in published maps and institutional affiliations.

## Supplementary Material

Supplementary Figure 1

## Figures and Tables

**Table 1:** Minimum Inhibitory Concentrations (MICs) of D-form and L-form KLKLLLLLKLK-NH_2_ against *S. aureus, E. coli*, and *C. albicans*.

	MIC (μg/ml)	
	D-form	L-form
*S. aureus*	1	16
*E. coli*	8	16
*C. albicans*	32	64

**Table 2:** Minimum Inhibitory Concentrations (MICs) of D-form and L-form antimicrobial peptides against *S. aureus*.

	MIC (μg/ml)	
	D-form	L-form
KLKLLLKLK-NH_2_	4	>128
FIKRIARLLRKIF-NH_2_	4	4
INLKAIAALAKKLL-NH_2_	16	16
FLPLIGRVLSGIL-NH_2_	8	8
