# Supplementary material for: D-form KLKLLLLLKLK-NH2 peptide exerts higher antimicrobial properties than its L-form counterpart via an association with bacterial cell wall components
Source: Sci Rep. 2017 Mar 6;7:43384. doi: 10.1038/srep43384 (PMC5338256; doi:10.1038/srep43384)
Supplement: Supplementary Figure 1 [file srep43384-s1.pdf]

D-form KLKLLLLLKLK-NH2 peptide exerts higher antimicrobial properties than its L-form counterpart via an association with bacterial cell wall components

Takayuki Manabe and Kiyoshi Kawasaki#

Supplementary information

a

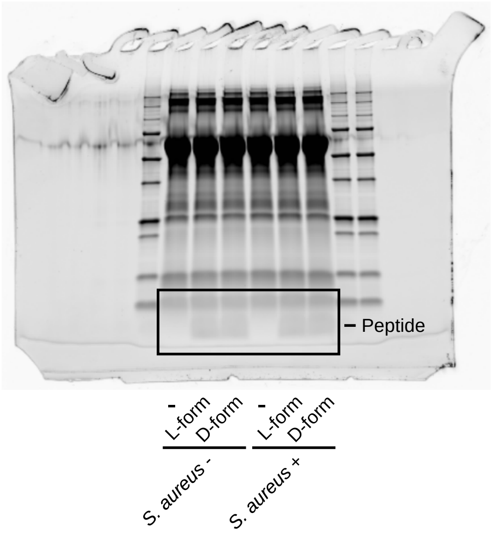

b

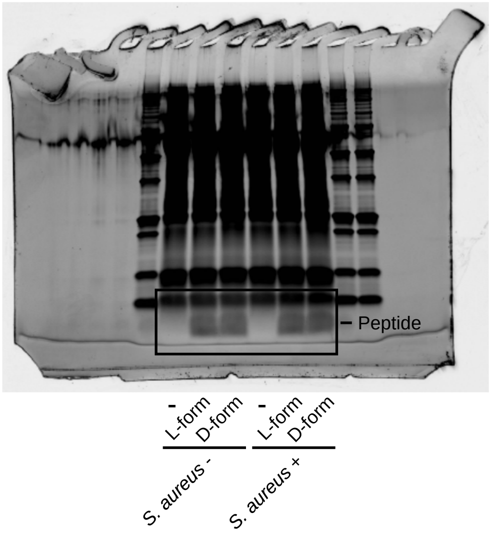

Supplementary Figure 1. Multiple contrast images of full-length gel for Figure 1g.

Figure 1g was prepared from image b.
